# Presenting and Evaluating a Smartwatch-Based Intervention for Smoking Relapse (StopWatch): Feasibility and Acceptability Study

**DOI:** 10.2196/56999

**Published:** 2024-11-21

**Authors:** Chris Stone, Rosie Essery, Joe Matthews, Felix Naughton, Marcus Munafo, Angela Attwood, Andy Skinner

**Affiliations:** 1 School of Psychological Science University of Bristol Bristol United Kingdom; 2 Integrative Cancer Epidemiology Programme University of Bristol Bristol United Kingdom; 3 MRC Integrative Epidemiology Unit University of Bristol Bristol United Kingdom; 4 School of Primary Care, Population Sciences and Medical Education University of Southampton Southampton United Kingdom; 5 Addiction Research Group University of East Anglia Norwich United Kingdom; 6 Bristol Medical School University of Bristol Bristol United Kingdom

**Keywords:** smoking, smoking cessation, passive detection, just-in-time intervention, JITAI, relapse prevention, relapse, smartwatch, wearable technology, wearable, mobile health, mHealth, mobile phone

## Abstract

**Background:**

Despite the benefits of smoking cessation, maintaining abstinence during a quit attempt is difficult, and most attempts result in relapse. Innovative, evidence-based methods of preventing relapse are needed. We present a smartwatch-based relapse prevention system that uses passive detection of smoking to trigger just-in-time smoking cessation support.

**Objective:**

This study aims to evaluate the feasibility of hosting just-in-time smoking cessation support on a smartwatch and the acceptability of the “StopWatch” intervention on this platform.

**Methods:**

The person-based approach for intervention development was used to design the StopWatch smoking relapse prevention intervention. Intervention delivery was triggered by an algorithm identifying hand movements characteristic of smoking from the smartwatch’s motion sensors, and the system-generated intervention messages (co-designed by smokers) were delivered on the smartwatch screen. A total of 18 smokers tested the intervention over a 2-week period, and at the end of this period, they provided qualitative feedback on the acceptability of both the intervention and the smartwatch platform.

**Results:**

Participants reported that the smartwatch intervention increased their awareness of smoking and motivated them to quit. System-generated intervention messages were generally felt to be relevant and timely. There were some challenges with battery life that had implications for intervention adherence, and the bulkiness of the device and the notification style reduced some participants’ acceptability of the smartwatch platform.

**Conclusions:**

Our findings indicate our smoking relapse prevention intervention and the use of a smartwatch as a platform to host a just-in-time behavior change intervention are both feasible and acceptable to most (12/18, 66%) participants as a relapse prevention intervention, but we identify some concerns around the physical limitations of the smartwatch device. In particular, the bulkiness of the device and the battery capacity present risks to adherence to the intervention and the potential for missed detections. We recommend that a longer-term efficacy trial be carried out as the next step.

## Introduction

### Smoking Cessation

Smoking is the primary cause of preventable illness and premature death, harming nearly every organ of the body and reducing both quality of life and life expectancy [1]. Tobacco use kills >7 million people each year [2], with a further 1.2 million deaths caused by exposure to secondhand smoke. Smoking is described by the World Health Organization as “one of the biggest public health threats the world has ever faced” [3]. For the individual, stopping smoking brings several health benefits, some relatively quickly and others on a more sustained basis as smoking cessation continues [4].

Smoking cessation interventions may include nicotine replacement therapy, prescription medication, and behavioral support through counseling by health care professionals. The standard treatment program recommended by the UK National Centre for Smoking Cessation and Training consists of a prequit assessment (1 or 2 weeks before an agreed quit date) followed by weekly counseling sessions until 4 weeks after the quit date [5]. At the prequit assessment, nicotine replacement therapy, other stop-smoking medications, and vaping are discussed. Nicotine replacement therapy is available in many forms, such as patches, nasal sprays, gum, or lozenges and may be used as a combination of a patch with another product. Other medications that may be taken are varenicline, taken for 12 weeks, or bupropion, taken for 7 to 9 weeks, both starting 1 week before the quit date. Basic guidance may also be given on how to use a vaping device (e-cigarette) and its use in combination with nicotine replacement therapy. Despite the support offered, as many as 75% of smokers who are abstinent for 4 weeks after their quit date will relapse to smoking within 1 year [6]. Therefore, the provision of additional support when the smoker lapses would clearly be beneficial as an adjunct to these more conventional therapies, as a means of improving adherence and ultimately the success of the quit attempt.

### Digital Interventions

The widespread use of the internet and the ubiquity of smartphone ownership have enabled new opportunities to reach smokers and provide them with smoking cessation support. This can be achieved through a variety of tools, such as SMS text messaging, automated emails, web-based self-help, internet-delivered counseling, and mobile apps. A recent review showed that digital-based smoking interventions can increase both short-term and sustained quit rates compared to traditional interventions [7]. A review of randomized controlled trials comparing automated digital interventions to self-help guidelines found that digital interventions had a clear positive effect on smoking cessation and that their effectiveness was associated with the extent to which the intervention was based on psychological theory-related constructs [8]. The value of using psychological behavior change techniques is further demonstrated in a study exploring the acceptability of an app (Quit Genius) based on cognitive behavioral therapy (CBT) and a non–CBT-based app (National Health Service Smokefree); users of the CBT-based app reported increased motivation to quit and a greater willingness to continue using the app [9]. Other studies also suggest that digital smoking cessation interventions can improve prolonged abstinence rates [10,11].

### Just-in-Time Smartwatch Interventions

For those trying to give up smoking, an initial lapse (ie, a single or time-limited episode of smoking) is a strong predictor of subsequent full relapse to smoking (ie, a resumption of smoking and the end of an attempt to quit), particularly during the first 2 weeks of a quit attempt [12]. Therefore, if the point of lapse can be identified and an intervention is delivered at that point, there is an opportunity to reduce the risk of relapse. Mobile and sensor technologies make this possible and targeting the progression from lapse to relapse rather than just trying to prevent lapses is a practical and potentially effective approach [13]. This could be achieved using a “just-in-time adaptive intervention,” an intervention design that adapts the provision of support to an individual’s changing context, with the aim of delivering that support at the moment and in the context that the person needs most [14,15].

Existing smoking cessation just-in-time adaptive interventions, such as Quit Sense [16], have been based on smartphones and have demonstrated clear benefits in helping with smoking cessation. One area in which there is scope for improving these interventions is the automation of user input. Currently, these interventions rely on the user manually registering when they smoke, which places a burden on the smoker. An improvement would be to make an intervention that is as close as possible to being fully automatic and that requires no input from the user to register smoking events. Recent developments in wearable technology have enabled this approach to be implemented on smartwatches. While there are some drawbacks with the use of these devices, such as the need for the user to wear and keep an additional device charged, smartwatches offer several potential advantages over smartphones in the context of these types of interventions. Most importantly, system-generated intervention messages are more easily seen at the time they are delivered on a smartwatch than on a smartphone. Despite the widespread belief that smartphones are always at hand, they may actually only be within reach around 50% of the time [17,18], whereas smartwatches, as long as they are worn, are never more than a glance away.

### This Study

In this study, we explored the feasibility and acceptability of what we believed to be the first smartwatch-based, just-in-time adaptive intervention for preventing smoking relapse. The platform for this intervention is the StopWatch system for passive detection of smoking, described in our earlier paper [19]. This system, an open-source smartwatch app developed by our team, uses an algorithm to process data captured by the motion sensors (accelerometer and gyroscope) of a commercially available smartwatch to identify signature gestures of cigarette smoking. Detection of smoking then triggers the delivery of an app-generated text message by the smartwatch to the smoker at the point of lapse. The intervention uses the person-based approach as its design methodology (described in the *Methods* section) to ensure that the design objectives and key features of the intervention are based on the intervention users’ needs.

Therefore, the aims of this study are (1) to evaluate the feasibility of using a smartwatch to host just-in-time smoking cessation support and (2) to assess the acceptability of the “StopWatch” smoking relapse prevention intervention as delivered on a smartwatch platform

## Methods

### Ethical Considerations

As this research involved human participants, ethics approval was obtained from the School of Psychological Science Research Ethics Committee at the University of Bristol (as per institutional guidelines), who reviewed this study and issued the approval code 12067 to confirm formal ethics approval. Prospective participants were provided with information explaining the nature, purpose, and risks of the study and were given opportunity to raise any questions with the investigators before making a decision to participate. Informed consent was obtained from participants by means of a web-based consent form. Participants were also informed that they were free to withdraw from the study at any time if they so wished, without needing to give any reason for withdrawing. The data obtained from participants has been anonymized and they are not identifiable. At the end of their time in the study, participants received compensation of £50 (US $63 at the time of the study) and could choose to receive this either by bank transfer or in the form of shopping vouchers.

### Intervention Design

This intervention was developed using the person-based approach, a proven approach for designing and optimizing behavior change interventions that places its intended users’ needs and preferences at the center of the planning and development process [20]. This initially involved drawing on existing qualitative evidence about individuals’ experiences of making and sustaining quit attempts, alongside input from patient and public contributors. This informed the development of a set of “guiding principles.” These are key, context-specific behavioral issues relevant to the intervention’s intended users that help ensure that its content and functionality are as persuasive, relevant, and engaging as possible. The guiding principles comprise a set of design objectives and the key features that address these objectives (Multimedia Appendix 1).

Our person-based approach guiding principles identified that a key component of the intervention should be brief smartwatch text messages delivered when smokers experience a smoking lapse that encourage continued smoking abstinence. These brief app-generated text messages will be delivered on the same smartwatch that is running the StopWatch detection system.

For our intervention, the design objectives were identified as (1) to challenge problematic cognitions (eg, inaccurate beliefs about smoking and fatalistic thinking) to empower users to continue their cessation attempt despite the lapse and reduce feelings of helplessness or lack of control, (2) to facilitate the management of difficult emotions and affective experiences related to quitting smoking at the point of lapse, (3) to offer a sense of connection to support, and (4) to facilitate avoidance of returning to habitual behaviors. Features of the StopWatch intervention that address these design objectives include smartwatch text messaging framed around the benefits of being smoke-free as opposed to the risks of continuing to smoke; using terminology preferred by smokers; delivering encouraging, reassuring, supportive, and nonjudgmental smartwatch text messages; and alerting on the detection of smoking to heighten awareness and reduce automaticity of behavior.

Another person-based approach component is the preliminary intervention logic model. This model hypothesizes the expected mechanisms through which the planned intervention is expected to have its intended effects and maps the intervention aims onto outcomes via behavioral determinants, intervention components, intervention processes, and behavior change mechanisms. The logic model for our intervention is provided in Multimedia Appendix 2.

### Intervention Platform

The smartwatch used in this feasibility evaluation is a commercially available device, the Ticwatch C2 Android smartwatch, manufactured by the Mobvoi Information Technology Company Limited. This device uses Google’s “Wear OS” smartwatch operating system. The StopWatch system for passive detection of cigarette smoking was validated on this device before its use in this study [21]. Validation consisted of initially observing the smokers’ eating, drinking, and smoking behaviors in the laboratory, followed by measuring their cigarette smoking over a 24-hour period in free-living conditions [19]. This free-living smoking measurement was subsequently repeated with the specific smartwatch model used in this study [21], recording the number of correct, false-positive, and false-negative detections to calculate estimates of sensitivity and specificity.

The starting point for the passive detection (ie, detection by sensors without requiring input from the participant) of cigarette smoking is raw data captured from the smartwatch accelerometer and gyroscope. Data falling below a predetermined threshold are filtered out to remove unwanted noise, and the salient motion features for forearm and hand movements are extracted. A decision tree then classifies the motion features, identifying signature gestures of smoking and applying rules to determine instances of cigarette smoking. Detection of an instance of smoking, which is a lapse in the smoker’s quit attempt, acts as a trigger for the delivery of the just-in-time intervention. In terms of the performance of the detection system, the sensitivity (the percentage of smoking incidents detected) was 78% and the specificity (the percentage of smoking incidents detected that were actually smoking) was 88% [21].

Our choice of smartwatch device (Mobvoi Ticwatch C2) was determined by the need to use a smartwatch that had a good battery capacity, was sufficiently robust to cope with use in participants’ daily lives, had adequate on-board data storage, and was available at a price point (£180, US $226 at the time of the study) that was realistic when purchasing multiple devices for use in a study. Importantly, the smartwatch also had to be an Android device as the detection mechanism was developed for this operating system due to it being open source. This model was typical of smartwatches commercially available at the time the study was conducted and remains representative of devices available at the time of writing.

### Intervention Smartwatch Text Messages

**Figure 1:**
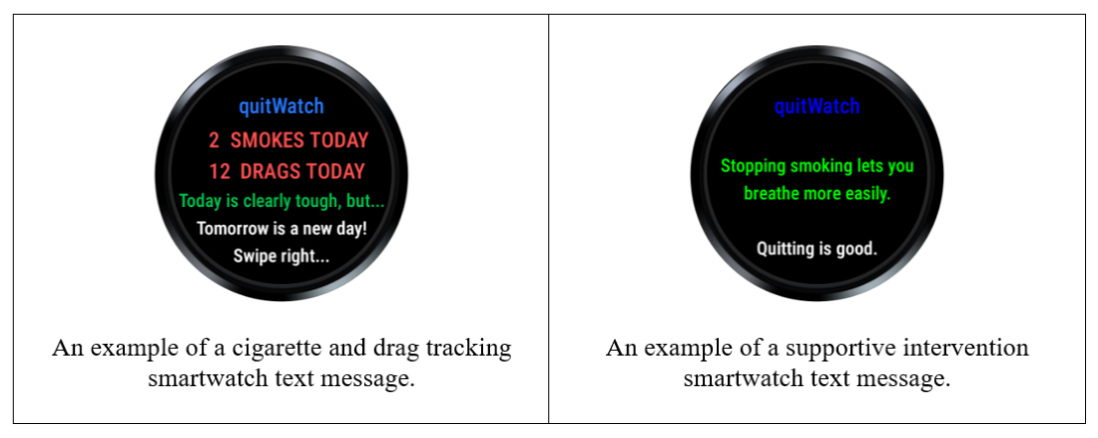
Example intervention screenshots.

The intervention itself consists of supportive stop-smoking app-generated text messages delivered on the smartwatch screen at the point of lapse, which are intended to prevent the lapse from becoming a full relapse to habitual smoking. The intervention smartwatch text messages displayed to the smoker were developed in collaboration with another study at the University of Bristol that had produced a suite of smoking-related support messages co-designed with input from smokers and former smokers [22]. Screenshots of app-generated intervention text messages displayed on the smartwatch are provided in Multimedia Appendix 3 (some examples are presented in Figure 1). For the purposes of evaluating the feasibility of the intervention, participants received one of a sample of 4 smartwatch text messages or a smartwatch text message alerting them to the number of cigarettes smoked and total number of drags taken so far that day. Selection of the smartwatch text messages was weighted such that there was a 40% probability of receiving the total cigarettes and drags smartwatch text message and a 60% probability of receiving one of the supportive smartwatch text messages (15% probability for each of these smartwatch text messages). Delivery of the smartwatch text message was via the smartwatch screen, and participants were alerted by means of a haptic vibration when a smartwatch text message was displayed on the screen. After reading the smartwatch text message, participants could dismiss it by swiping it away. In addition, for signposting to immediate support at any time, pressing a button on the smartwatch screen displayed information on accessing the UK National Health Service smoking cessation support web pages.

### Procedure

Participants were mailed a smartwatch programmed with the StopWatch intervention software, instructed on its use by means of a training video, and asked to wear the smartwatch over a 2-week period. During this time, participants went about their normal life, wearing the smartwatch from the moment they woke up until they went to bed when they were instructed to place the smartwatch on its charging cradle to recharge the battery overnight. Participants were advised to remove the smartwatch while showering, swimming, or performing any activities that might damage the device in other ways (eg, contact sports) and replace it on the wrist afterward.

At the end of the 2 weeks, participants mailed the smartwatch back to the researcher and were asked to provide qualitative feedback through a Qualtrics (Qualtrics International, Inc) [23] web-based questionnaire (Multimedia Appendix 4). Data captured by the StopWatch system were downloaded to a secure server and deleted from the smartwatch. Participants who completed the study were reimbursed £50 (US $63 at the time of the study) for their time.

### Adherence and Acceptability

Adherence and acceptability were assessed using a Qualtrics questionnaire consisting of 27 questions to elicit participant feedback, with both closed-ended (yes or no or Likert scale) and free-text responses. Of these 27 questions, 14 (52%) were related to the smartwatch itself, 11 (41%) questions were related to the intervention, and 2 (7%) questions allowed the participants to provide more general feedback about the study.

Participant responses to the free-text questions were independently analyzed for themes by authors CS and AS. There was a high level of concordance between the themes identified, which were then used as thematic codes against which each participant’s responses were coded. Some responses are also quoted verbatim at the end of the *Results* section.

### Participants: Recruitment

Participants were recruited using the University of Bristol Tobacco and Alcohol Research Group website and digital newsletter and through a University of Bristol social media channel primarily used by nonacademic staff. However, as with participant recruitment at many universities, recruitment through the University of Bristol channels typically reaches a higher-educated, higher socioeconomic position demographic, so to balance this, we also recruited through other channels aimed at lower socioeconomic positions and under-served groups. To achieve this, we enlisted the services of a web-based marketing agency [24]. The marketing agency organized a multichannel campaign consisting of web-based recruitment advertisements published on a local news media website (for 2 weeks) and on websites using Google Display Network (for 6 weeks), both targeted to audiences from lower socioeconomic position areas of the city of Bristol and print-media recruitment advertisements published on the front pages of 2 local newspapers on 3 days over a 2-week period. In addition, the study was promoted through local community-based health provider networks in inner-city areas of east and south Bristol.

Socioeconomic position was determined by postcodes corresponding to electoral wards, categorized according to levels of deprivation in a report produced by the Bristol City Council, the University of Bristol, and the University of the West of England [25]. The socioeconomic position is described as high for neighborhoods where the proportion of residents with low income is <10%, moderate for those where it is between 10% and 19%, and low for those where ≥20% of residents have low income. The measure of low income used is the Index of Multiple Deprivation, as defined by the UK Department for Communities and Local Government [26]. This is the most widely used index used by the UK Government to measure the proportion of the population experiencing low income in small areas in England, and it is calculated using several indicators related to income and the receipt of state benefits.

To be included in the study, participants were required to be UK residents aged between 18 and 70 years, daily cigarette smokers actively seeking to quit (smoking ≥10 cigarettes per day), and smokers who habitually smoke with their right hand. Persons with any mobility issues affecting their right hand or right arm and users of e-cigarettes or any form of nicotine replacement therapy during the study period were excluded from taking part in this study.

The protocol for this study was preregistered on the Open Science Framework [27].

## Results

### Participants: Demographics

A total of 31 people responded to the study advertisements and 21 (68%) were recruited, with 5 (16%) not meeting the inclusion criteria and 5 (16%) signing up but withdrawing before the start of the study. In addition, 1 (3%) participant did not start data collection due to a faulty smartwatch, and this participant was excluded to give a total of 20 participants. The characteristics of these participants are presented in Table 1.

**Table 1:** Participant characteristics (n=20).

Participant characteristic				Participants
**Sex, n (%)**				
	Female			13 (65)
	Male			7 (35)
**Ethnicity, n (%)**				
	**Asian (this participant was Chinese)**			1 (5)
	**Black or Black British or Caribbean**			1 (5)
	**Middle Eastern (this participant was Iranian)**			1 (5)
	**White**			17 (85)
		White British	6 (30)	
		White English	6 (30)	
		White French	1 (5)	
		White Irish	1 (5)	
		White Spanish	1 (5)	
		White others	2 (10)	
**SEP^a^, n (%)**				
	High SEP neighborhood			7 (35)
	Moderate or mixed SEP neighborhood			6 (30)
	Low SEP neighborhood			7 (35)
**Age (y), mean (SD)**				
	Overall			37.5 (16.09)
	Female			41 (17.05)
	Male			34 (13.18)

^a^SEP: socioeconomic position.

### Adherence and Acceptability: The Intervention

In total, 2 (10%) of the 20 participants did not complete the study. Of these, 1 (5%) declined to provide feedback, and 1 (5%) failed to provide feedback or return the smartwatch and ceased responding to any communication from the study team.

For the remaining 18 participants, during their 14-day study period, the self-reported median wear time for the smartwatch was 12.8 (IQR 12-14) days. The median wear time each day (participant self-report) was 10.6 (IQR 10-12) hours (n=17, 94%). The reasons for not wearing the smartwatch are presented in Table 2. The participants were able to enter free text identifying >1 factor; hence, the number of factors is higher than the number of participants (n=7, 39% of the participants identified 1 factor only and n=11, 61% identified >1 factor).

**Table 2:** Adherence to the intervention based on self-reported factors affecting the amount of time the smartwatch was worn.

Adherence factors			Participants (n=18), n (%)
**Reasons for not wearing the smartwatch at any time (factors identified by participants) taken from response to question 4, “On the occasions where you didn’t wear the smartwatch, why was this? (For example, did you find the watch uncomfortable to wear? Did the battery run down so you took it off to charge it? Did you participate in activities where wearing the watch was impractical, such as sports?).” Free-text responses are classified into themes.**			
	Short battery life	11 (61)	
	Incompatible with activity (eg, at work and while playing a sport)	8 (44)	
	Removed while sleeping	5 (28)	
	Removed while showering	4 (22)	
	Discomfort	3 (17)	
	Appearance	1 (6)	

### Adherence and Acceptability: The Smartwatch Platform

One-third (6/18, 33%) of the participants reported that the watch was uncomfortable to wear (5/18, 28% of the participants stated that this was due to the watch’s bulkiness and 1/18, 6% stated that this was due to the fit of the watchstrap). Between one-third and half of the participants (7/18, 39%) reported that the smartwatch helped increase their awareness of smoking, although 1 (6%) participant reported finding the smartwatch to be a negative reminder about smoking. The positive and negative self-reported factors relating to acceptability are provided in Table 3.

**Table 3:** Acceptability of the smartwatch platform in terms of comfort and positive and negative aspects of wearing and using the smartwatch.

Acceptability factors		Participants (n=18), n (%)
**Comfort of wear (participant ratings are based on question 5, “How comfortable was the smartwatch to wear?” and include response options)**		
	Very comfortable	0 (0)
	Somewhat comfortable	9 (50)
	Neither comfortable nor uncomfortable	3 (17)
	Somewhat uncomfortable	4 (22)
	Very uncomfortable	2 (11)
**Positive aspects of wearing and using the smartwatch (factors identified by participants are based on question 14, “Please describe any positive aspects of wearing and using the smartwatch,” and free text responses are classified into themes)**		
	Increased awareness of smoking	7 (39)
	Ability to track smoking	6 (33)
	Comfort and light weight	3 (17)
	Helpful smartwatch text messaging	2 (11)
	Other functionality of smartwatch	2 (11)
	Discreet format of device	1 (6)
**Negative aspects of wearing and using the smartwatch (factors identified by participants are based on question 15, “Please describe any negative aspects of wearing and using the smartwatch,” and free text responses are classified into themes)**		
	Inaccurate detections	6 (33)
	Haptic prompts accompanying smartwatch text messages were too intense	5 (28)
	Bulkiness, weight, and discomfort	3 (17)
	Short battery life	3 (17)
	Risk of damage when playing a sport	1 (6)
	Demands of keeping the smartwatch charged	1 (6)
	Limited functionality as a smartwatch	1 (6)
	Reminder about smoking	1 (6)
	Device was indiscreet (drew attention to quit attempt)	1 (6)

### Adherence and Acceptability: The Charging Regime

Most (16/18, 89%) participants experienced times when the battery ran out. When asked why they thought this might be, 4 (22%) out of 18 participants identified excessive movement (and therefore, higher sensor demand), 4 (22%) suggested a lack of battery capacity, 2 (11%) suggested potentially high power demands of the vibration alerts, 2 (11%) indicated that they did not adhere to a regular charging regime, and 1 (6%) thought that it could be due to longer wear time. Participants who experienced a problem keeping the smartwatch battery charged indicated that this was mainly due to the battery running down quickly (4/18, 22%), the smartwatch running out of charge at an inconvenient time (1/18, 6%), the urgency of the notifications to recharge the battery when at low power (1/18, 6%), and the battery life being shorter than expected may be due to the screen being too bright (1/18, 6%).

Responses to questions relating to adherence and acceptability of the charging regime are provided in Table 4.

**Table 4:** Adherence to and acceptability of the charging regime.

Smartwatch battery charging				Participants (n=18), n (%)
**Adherence**				
	**Did you place the smartwatch on its charger overnight? (question 9 and response options yes or no)**			
		Yes	18 (100)	
		No	0 (0)	
	**Did charging the watch overnight give you sufficient power for a day’s use? (question 10 and response options yes or no)**			
		Yes	7 (39)	
		No	11 (61)	
	**Did you find there were times when the battery ran out? (question 12 and response options)**			
		No	2 (11)	
		Sometimes	9 (50)	
		Often	7 (39)	
**Acceptability**				
	**How much of a problem was it to keep the smartwatch charged? (question 7 and response options)**			
		None at all	2 (11)	
		A little	7 (39)	
		A moderate amount	5 (28)	
		A lot	4 (22)	
		A great deal	0 (0)	

### Acceptability: Smartwatch Text Messaging

Table 5 presents participants’ ratings of the timing and content of the smartwatch text messages. Only 3 (17%) of the 18 participants felt that the timing of smartwatch text message delivery was inappropriate; this was felt to be the case for 2 (11%) participants. This was because a smartwatch text message was delivered when the participant was not smoking (false positive), and 1 (6%) participant felt the smartwatch text message was delivered too late.

**Table 5:** Participant ratings of smartwatch text messaging regarding the timing, content, and helpfulness of the smartwatch text messages.

System-generated intervention messaging			Participants (n=18), n (%)
**Timing of** **smartwatch text** **message** **delivery (question 16, “How would you rate the timing of the intervention message delivery?” and response options)**			
	Very appropriate	4 (22)	
	Somewhat appropriate	8 (44)	
	Neither appropriate nor inappropriate	3 (17)	
	Somewhat inappropriate	1 (6)	
	Very inappropriate	2 (11)	
**Content of** **system-generated intervention messages** **(question 18, “How would you rate the content of the intervention messages?” and response options)**			
	Very relevant	2 (11)	
	Somewhat relevant	9 (50)	
	Neither relevant nor irrelevant	5 (28)	
	Somewhat irrelevant	1 (6)	
	Very irrelevant	1 (6)	
**Did you find any of the intervention messages particularly helpful? (question 20 and response options)**			
	Yes	4 (22)	
	No	5 (28)	
	Maybe	9 (50)	

Most (11/18, 61%) participants reported that the content of the smartwatch text messages was relevant to them, with only 2 (11%) participants reporting it as not relevant. Where the content of the smartwatch text messages was found to be irrelevant, this was further described by participants as the smartwatch text message being patronizing and uninformative or unhelpful (1 participant each).

When asked how the intervention aligned with the participants’ quit attempts, positive responses were that it raised awareness about smoking, it made them feel positive about quitting, it made them smoke slightly less, it made them stop and think, it provided constant encouragement, and it warned of high smoking. Negative responses were that repeated smartwatch text messages lost effectiveness, the message did not appear early enough during smoking, messages lacked variation and some were vague, and the support signposting to the National Health Service website did not function as a link. Encouraging smartwatch text messages were identified as being most helpful (3/18, 17%) followed by messages that tracked smoking (2/18, 11%). Factual smartwatch text messages about smoking and cessation, warning messages, motivational messages, messages that were reassuring, and messages that were supportive were identified as helpful by one participant each. Participants generally found the smartwatch text messages helpful; when asked whether they found any of the messages unhelpful, 6 (33%) of the 18 participants responded “yes” and 12 (67%) responded “no.” Reasons given for smartwatch text messages being found unhelpful were that the messages were repetitive (2/18, 11%), generic (1/18, 6%), inaccurate (1/18, 6%), chastising (1/18, 6%), and became familiar after repeated presentation (1/18, 6%).

### Overall Acceptability

Positive aspects and negative aspects of participants’ experience with the intervention included are presented in Textbox 1.

**Quotes describing positive and negative aspects of participants’ experience with the intervention.**

**Illustrative quotes describing positive aspects of participants’ experience**

- “It made me more mindful of when I was smoking.”
- “Made me rethink my relationship with smoking...pushed me to start cutting down.”
- “It made me think about how much I was smoking during the day, and I have slightly cut down as a result.”
- “It constantly makes you aware that you are doing something your body craves but your mind wants to quit—this is a good incentive to not smoke.”
- “Having it on reminded me of not smoking.”
- “I generally don’t tend to realise I am craving a cigarette before I light up - the watch made me more aware of this subconscious habit.”
- “It made me and those around me aware of my aim to stop smoking.”
- “It made me aware of how many cigarettes I have a day.”

**Illustrative quotes describing negative aspects of participants’ experience**

- “Too heavy, too noisy, too bright, misreading my arm movements”
- “It didn’t always pick up smoking occurrences and didn’t always recognise when I’d finished smoking if I went on to other activities using my right arm.”
- “The battery would not last an entire day, it was impractical to wear it while doing sports, it would sometimes vibrate very loudly when charging.”
- “It was quite big and the recharge warning was very disturbing, for example when it went off at work.”
- “Vibrations were perhaps too intense when it wanted to notify me of something.”
- “False positives made me think of smoking.”
- “Have to charge it every night and take care of charging.”
- “People asking about it, why it’s flashing and what’s the time and I had to look at my phone.”

## Discussion

### Principal Findings

In this study, we explored the feasibility and acceptability of using a smartwatch-based, just-in-time adaptive intervention to prevent smoking lapses, which could lead to full relapse to smoking, in smokers motivated to quit. We achieved this by measuring adherence to the intervention, exploring the acceptability of the smartwatch as a platform for delivering the intervention, and exploring the acceptability of smartwatch text messages. Our findings, which are discussed in more detail in the subsequent sections, reveal that the use of a smartwatch to host a just-in-time smoking relapse prevention intervention is both feasible and acceptable to most (12/18, 66%) participants, with adherence being comparable to other applications of smartwatches in health research. Most (12/18, 66%) participants found the smartwatch text messages timely, and 61% (11/18) found them relevant; however, limitations of battery capacity and the physical size of the device presented challenges to the acceptability of the smartwatch-delivered intervention for some participants.

### Adherence

Adherence to wearing the smartwatch, with mean wear times of 12.77 (SD 2.42) out of 14 days and 10.62 (SD 2.03) hours per day, compares well with other studies using consumer smartwatches for health research. Galarnyk et al [28] found that participants wore smartwatches recording physical activity and heart rate for 5.3 days per week, while Beukenhorst et al [29], in a study measuring physical activity and self-reports of arthritis symptoms, found a daily wear time of 11.2 hours. All participants charged the smartwatch overnight each night during the study period, representing 100% (18/18) adherence to the required charging regime. However, the relatively short battery life was a major cause of the watch not being worn, with more than half (11/18, 61%) of the participants having to remove the smartwatch to recharge the battery during the day. This is consistent with other investigations using smartwatches to collect sensor data. For example, Rouzaud Laborde et al [30] found that the lack of battery life significantly impacted engagement with their smartwatch app for capturing self-report data on osteoarthritis symptoms. Similarly, another study by Beukenhorst et al [31] made the point that improvements in battery life are needed for continuous data collection of high-frequency sensor data, while in a systematic review of smartwatch uses for health and wellness [32], limited battery power was identified as a commonly reported issue in smartwatch studies. The need to recharge the smartwatch during the day could be a severe limitation to the effectiveness of the intervention, as some smoking instances could be undetected, resulting in missed opportunities to intervene. Having to remove the smartwatch for activities such as sports, swimming, and other situations where the smartwatch could become damaged also reduces the potential for adherence to the intervention. In our study, participants were advised to remove the smartwatch during activities such as showering, swimming, and playing contact sports to avoid damaging the device. A more robust type of smartwatch than our evaluation model (which is not designed for use in physically challenging conditions and has an ingress protection rating of IP68 [33], meaning that it is resistant to water to some degree but is not fully waterproof) could perhaps improve adherence in these contexts.

We have used self-reported measures of wear time. Ideally, wear-time metrics would be objective, sensor-based measures that are not subject to recall and other biases. We did attempt this using a number of different sensors and approaches but were not able to arrive at an acceptable solution that provided an accurate measure of wear time without significantly impacting battery life. Therefore, dependence on self-report as a measure of wear time is a limitation of this study.

### Intervention Smartwatch Text Messaging

In terms of the intervention itself, 66% (12/18) of the participants rated the timing of smartwatch text message delivery as appropriate, and 61% (11/18) rated the content of smartwatch text messages as relevant. This compares well with the findings of the feasibility trial of Quit Sense [16], a smartphone app that uses location sensing to trigger just-in-time smoking cessation support, in which 67% of the participants were sufficiently satisfied with the intervention to be able to say that they would recommend it to a friend. However, 16% (3/18) of the participants found the smartwatch text message timings in our intervention inappropriate. Timing issues could have arisen from inaccurate (false positive) detections, and content issues may be due to use of a smaller subset of smartwatch text messages in our feasibility evaluation.

The tone of the smartwatch text messages is also important. A total of 10% (2/18) of our participants found the message content irrelevant. However, the smartwatch text message styles that were encouraging and supportive were found to be helpful, which reflects the design objectives contained in the guiding principles we developed using the person-based approach.

Another design objective was alerting the user upon detection of smoking action to heighten awareness and reduce the automaticity of behavior, and this was identified by participants as helping to align with their quit attempt. However, it has to be noted that some (5/18, 28%) participants found the smartwatch text messages to be unhelpful and repetitive. In total, 28% (5/18) answered “no” to the question “Did you find any of the messages particularly helpful” and 50% (9/18) responded “maybe” to this question. This may be partly due to the small subset of smartwatch text messages used in this feasibility evaluation, causing the messages to be viewed more frequently. Using a larger set of smartwatch text messages would have resulted in more novel messages appearing, which may have elicited a more positive response from these participants.

We programmed the haptic vibration alerts to be very noticeable to minimize the risk of intervention users missing a smartwatch text message or a notification to recharge the battery. Some participants (5/18, 28%) found these haptic alerts too intrusive and, therefore, indiscreet. A study of emotional factors relating to smartwatches identified “device annoyance” as a potential barrier to continued smartwatch use [34], and in light of this and participants’ feedback, further work may be needed to identify the optimal balance between noticeability and subtleness of the smartwatch text message delivery mechanism.

In terms of the detection of smoking itself, 33% (6/18) of the participants quoted inaccurate detection as a negative aspect of using the watch, which is potentially a concern. Our previous validation study indicated that the StopWatch detection algorithm has a sensitivity of 78% (the percentage of smoking incidents detected) and a specificity of 88% (the percentage of detections that were actually smoking [21]. Further investigation of the participant data indicated that 2 (11%) of the 18 participants experienced a high number of false positives (which may have been triggered by other hand-to-mouth gestures, such as eating or drinking); however, most experienced 1 or none at all.

### Acceptability

Reports of comfort varied across participants, with 33% (6/18) rating it as “somewhat uncomfortable” or “very uncomfortable.” Some (3/18, 16%) participants found the bulkiness and weight of the smartwatch uncomfortable, although others (4/18, 21%) described this as a positive aspect. The perceived comfort of the smartwatch was important as this may reduce wear time and hence adversely impact adherence to the intervention. In one-third (6/18, 33%) of the participants who rated the smartwatch as “somewhat uncomfortable” or “very uncomfortable,” the mean number of days the watch was worn was slightly less compared to the whole sample (11.5 vs 12.8 days, respectively), as was the mean number of hours worn each day (9.5 vs 10.6 hours). Among those who specifically identified the bulkiness of the watch as the reason for its discomfort, the mean number of days the watch was worn was similar to the whole sample (12.8 vs 12.2 days, respectively), while the mean number of hours the watch was worn each day was more than the whole sample (11.5 hours vs 10.6, respectively). While comfort, in general, may reduce the time the watch is worn and therefore present a risk to adherence to the intervention, bulkiness itself does not appear to have this effect. It may also be the case that for some (4/18, 22%) participants, longer daily wear times increased the feeling of discomfort.

Of the 5 participants who described the smartwatch as being bulky, 4 (80%) were female individuals. This underlines the need to involve a broad range of potential participants when selecting devices for interventions of this kind.

The device’s appearance drew mixed reports; for example, one participant viewed the discreet appearance of the smartwatch as a positive characteristic, whereas another found that its presence drew attention to their quit attempt. Esthetic and subjective considerations may seem to be relatively trivial, but these may be important if they influence the uptake of smartwatch use and, consequently, the acceptability of the intervention.

### Practical Considerations

For pragmatic reasons, this feasibility evaluation only recruited participants who habitually use their right hand to smoke. Right-handedness for smoking (rather than general right-hand dominance) was specified in the inclusion criteria for the study, and participants were instructed to wear the smartwatch on their right wrist to ensure that the watch would be worn on the same hand as that used for smoking. People who smoke with their left hand would need to wear the smartwatch on their left wrist, which would reverse the motion sensor axes relative to hand movements, preventing the detection algorithm in its current form from operating correctly. For the intervention to be used in a general population, the smartwatch detection algorithm will need to cater to either handedness. In that case, it is likely that the user would need to select left-handed or right-handed orientation as a set-up action, which would configure the detection algorithm appropriately. Another consideration is that, in this study, wearing the smartwatch on the right hand may have been the opposite of some participants’ usual preference, and this may have contributed to some of the criticism of the watch’s comfort.

As an intervention delivery platform, the always-at-hand nature of smartwatches encourages a high degree of compliance, and this is particularly beneficial in the case of just-in-time interventions, where the key to the effectiveness of the intervention is the delivery of the right support at the right time. The trade-off between device size and battery capacity, where the battery has to be a certain size to achieve the level of performance required, is one of the challenges of smartwatch use and potentially a challenge to the implementation of interventions on a smartwatch as we have seen with participants’ experiences in this study. However, battery technology has improved considerably over time, and it is reasonable to assume that these improvements will continue. Advances in screen technology, such as active matrix organic light-emitting diode screens and memory-in-pixel displays (where a 1-bit memory circuit is embedded into every pixel enabling information to be retained), bring increased energy efficiency and extend battery life, although the higher cost of such devices could potentially make the intervention less cost-effective. In addition, smartwatches are increasingly being marketed as lifestyle products for sport performance monitoring and outdoor activities and so are being designed to be more water-resistant and durable. Improved battery life and durability will increase potential wear time, making smartwatches an even more attractive option for the intervention developer. In addition, ongoing enhancements to energy efficiency may reduce the need for a large battery and therefore enable the manufacture of smaller and more comfortable smartwatches, also increasing wear time and improving the potential for adherence to interventions.

### Limitations

It is important to note that this study is a relatively short-term study compared to the journey of a smoker who is trying to quit and is based on a small sample of participants. As mentioned earlier, our measures of wear time are self-reported. Crucially, we have not tested the efficacy of this intervention in improving quit rates or the success of smoking cessation attempts; it is recommended that further research be carried out to investigate this completely. This feasibility evaluation has used a limited subset of smartwatch text messages. We envisage that a larger trial of efficacy would use a much larger suite of smoking-related support smartwatch text messages such as the one co-designed with input from smokers and former smokers and developed by researchers at the University of Bristol [23].

### Conclusions

The findings from this study indicate that the use of a smartwatch to deliver just-in-time smoking relapse interventions is broadly feasible and acceptable to most (12/18, 66%) participants. However, participants identified some challenges too. In terms of the smartwatch platform, issues identified were battery life and comfort (factors such as weight and size). The design of the intervention itself is also an important consideration, with the nature of notifications (making the smartwatch text messaging noticeable without being indiscreet) and the timing and content of smartwatch text messaging being important factors identified by participants. At the heart of the intervention is the smoking detection algorithm, and the reliable operation of this algorithm is key to the delivery of intervention smartwatch text messages at the point when support is needed, and not at times that user may find them annoying, which may discourage the use of the intervention.

Despite these issues, the combination of sensors for passive detection, the capability for smartwatch text messaging and interactivity with the user, and the convenient wearable format makes smartwatches potentially powerful tools for delivering just-in-time interventions.

## Appendix

Multimedia Appendix 1 Person-Based Approach Guiding Principles.

Multimedia Appendix 2 Person-Based Approach Logic Model.

Multimedia Appendix 3 Intervention screenshots.

Multimedia Appendix 4 Qualtrics questionnaire for participant feedback.

## Abbreviations

CBT: cognitive behavioral therapy
