# Supplementary material for: Presenting and Evaluating a Smartwatch-Based Intervention for Smoking Relapse (StopWatch): Feasibility and Acceptability Study
Source: JMIR Form Res. 2024 Nov 21;8:e56999. doi: 10.2196/56999 (PMC11621715; doi:10.2196/56999)
Supplement: Multimedia Appendix 1 [file formative_v8i1e56999_app1.pdf]

Appendix 1 – Person-Based Approach Guiding Principles

Design objective	Key Features
To challenge problematic cognitions – e.g. inaccurate beliefs and attributions about smoking, and fatalistic thinking – to:<br><br>1) empower users to remain quit in the face of a lapse/relapse and<br>2) reduce feelings of helplessness/ lack of control	Messaging framed around benefits of remaining quit (especially short-term/immediate) as opposed to risks of continuing to smoke<br><br>Use of language/terminology preferred by target users/ avoidance of disliked terms<br><br>Present brief facts about benefits of remaining smoke free in terms of:<br>- immediate-term benefits (e.g. reduced breathlessness)<br>- benefits for treatment efficacy
To facilitate management of, and coping with, difficult emotions and affective experiences (e.g. cravings, self-blame, stress, anxiety, depression, feelings of isolation, irritability, restlessness, poor concentration; extreme emotions tied to quite attempt – both elation and defeat associated with getting through day without cigarette or failure to do so)	Signposting to external sources of support – e.g. websites<br><br>Encouragement to focus on the fact they are attempting to take action and making a positive decision for their future health<br><br>Reassurance that cravings and negative emotions are transient and will become less intense over time and eventually pass
To offer a sense of connection to a source of empathic/ non-judgemental support	Framing of messages in non-judgemental, autonomy supportive language<br><br>Messages acknowledging challenge of remaining smoke free and congratulating on small achievements<br><br>'Smoking detected' alerts accompanied by supportive message
To facilitate avoidance of returning to old routines and habitual behaviours	Alert presented on watch face when smoking detected to heighten awareness of behaviour and encourage self-monitoring/ reduce automaticity of behaviour
