# Supplementary material for: Presenting and Evaluating a Smartwatch-Based Intervention for Smoking Relapse (StopWatch): Feasibility and Acceptability Study
Source: JMIR Form Res. 2024 Nov 21;8:e56999. doi: 10.2196/56999 (PMC11621715; doi:10.2196/56999)
Supplement: Multimedia Appendix 2 [file formative_v8i1e56999_app2.pdf]

Appendix 2 – Person-Based Approach Logic Model

Intervention Aims	*Behavioural Determinants (TDF Domain Target)*	*Intervention Components (BCW intervention function)*	*Intervention Processes: BCTs*	*Mechanisms*	*Outcomes*
To reduce/prevent smoking relapse in smokers currently making an attempt to quit	**Contextual factors:**<br>- Duration of current quit attempt<br>- Previous quit attempts<br><br>**Psychosocial factors:**<br>- Beliefs about self and capability to remain abstinent/avoid relapse *(Beliefs about capability; Social role and identity)*<br>- Beliefs about smoking and consequences of smoking *(Beliefs about consequences; Knowledge)*<br>- Habitual/automatic nature of behaviour *(Behavioural regulation; Environmental context and resources; Social influences)*	**Smartwatch able to detect smoking action:**<br>- Real-time smoking detection alerts (*Education*)<br>- Summary statistics of daily smoking (*Education, Enablement*)<br>- Brief tips, informational and motivational support messages delivered at point of lapse (*Education, Persuasion, Environmental restructuring, Enablement, Incentivisation, Modelling)*	-Feedback on behaviour<br>-Information about antecedents<br>-Reattribution<br>-Information about health consequences<br>-Information about social and environmental consequences<br>-Information about emotional consequences<br>-Prompts/cues<br>-Behaviour substitution<br>-Habit reversal<br>-Graded tasks<br>-Social reward<br>-Reduce negative emotion<br>-Distraction	**Engagement**<br>- Engagement with/use of the smartwatch device<br>- Perceived utility and usability of the intervention<br><br>**Changes to determinants:**<br>- Decreased automaticity of smoking behaviour<br>- Increased self-efficacy<br>- Increased perceived importance/ value of remaining abstinent<br>- Increased intrinsic motivation to remain smoke-free<br><br>**Behaviours:**	**Primary Outcome:**<br>-Longer period of time before lapse<br>-Longer period of time before full relapse

	- Stress/anxiety/negative affect *(Emotion; Skills)*		-Valued self-identity<br>-Identity associated with changed behaviour<br>-Verbal persuasion about capability<br>-Focus on past success	-Longer periods between smoking<br>-Smaller number of smoking episodes	

TDF – Theoretical Domain Framework
BCT – Behaviour Change Technique
BCW - Behaviour Change Wheel (Michie et al, 2011[1])

1. Michie S, van Stralen MM, West R. The behaviour change wheel: A new method for characterising and designing behaviour change interventions. Implementation Sci. 2011; 6, 42. https://doi.org/10.1186/1748-5908-6-42 retrieved 24/08/2023, doi: 10.1186/1748-5908-6-42
