# Supplementary material for: Presenting and Evaluating a Smartwatch-Based Intervention for Smoking Relapse (StopWatch): Feasibility and Acceptability Study
Source: JMIR Form Res. 2024 Nov 21;8:e56999. doi: 10.2196/56999 (PMC11621715; doi:10.2196/56999)
Supplement: Multimedia Appendix 3 [file formative_v8i1e56999_app3.pdf]

Appendix 3 – Intervention screenshots

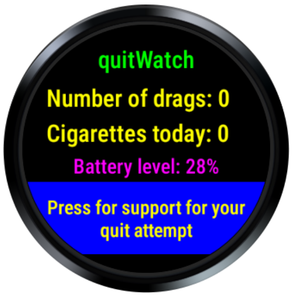

Intervention “home” screen

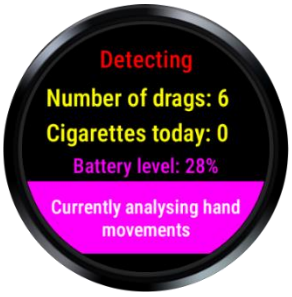

The display during the detection of smoking

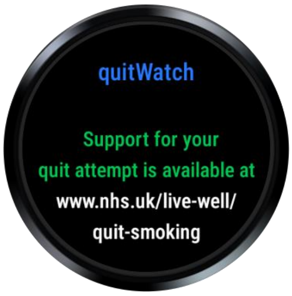

Signposting to support

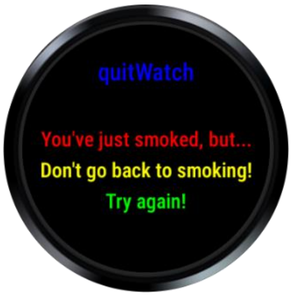

Intervention message (smartwatch text)

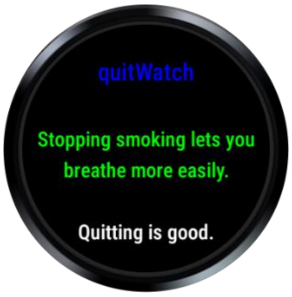

Intervention message (smartwatch text)

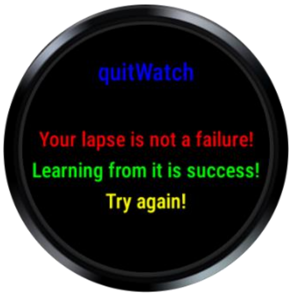

Intervention message (smartwatch text)

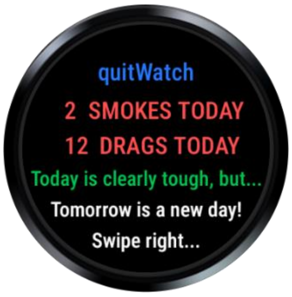

Tracking message (smartwatch text)

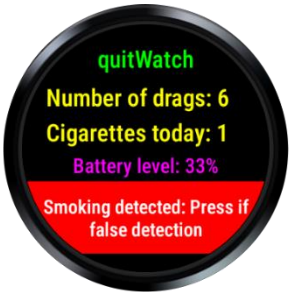

Option to remove false positive detection
