# Supplementary material for: Presenting and Evaluating a Smartwatch-Based Intervention for Smoking Relapse (StopWatch): Feasibility and Acceptability Study
Source: JMIR Form Res. 2024 Nov 21;8:e56999. doi: 10.2196/56999 (PMC11621715; doi:10.2196/56999)
Supplement: Multimedia Appendix 4 [file formative_v8i1e56999_app4.pdf]

## Appendix 4 – Qualtrics questionnaire for participant feedback

**Q1. Please enter your name.**
(Free text)

**Q2. How many days did you wear the smartwatch?**
(Free text)

**Q3. How many hours each day did you wear the smartwatch?**
(Free text)

**Q4. On the occasions where you didn't wear the smartwatch, why was this?**
*For example, did you find the watch uncomfortable to wear? Did the battery run down so you took it off to charge it? Did you participate in activities where wearing the watch was impractical (such as sports)?*
(Free text)

**Q5. How comfortable was the smartwatch to wear?**
(Very comfortable/Somewhat comfortable/Neither comfortable nor uncomfortable/Somewhat uncomfortable/Very uncomfortable)

If response to Q5 is "somewhat uncomfortable" or "very uncomfortable":
**Q6. What caused the smartwatch to be uncomfortable to wear?**
(Free text)

**Q7. How much of a problem was it to keep the smartwatch charged?**
(None at all/A little/A moderate amount/A lot/A great deal)

If response to Q7 is "a moderate amount" or "a lot":
**Q8. If charging the smartwatch was a problem, what was the cause?**
(Free text)

**Q9. Did you place the smartwatch on its charger overnight?**
(Yes/No)

**Q10. Did charging the smartwatch overnight give you sufficient power for a day's use?**
(Yes/No)

**Q11. Did anything prevent you from charging the smartwatch overnight?**
*For example, does your daily routine make it difficult to charge the smartwatch overnight (e.g. working nights)?*
(Free text)
**Q12. Did you find there were times when the battery ran out?**
(No/Sometimes/Often)

If response to Q12 is "sometimes" or "often":
**Q13. If there were times when the battery ran out, why do you think this happened?**
(Free text)

**Q14. Please describe any positive aspects of wearing and using the smartwatch.**
(Free text)

**Q15. Please describe any negative aspects of wearing and using the smartwatch.**
(Free text)

**Q16. How would you rate the timing of the intervention message delivery?**
(Very appropriate/Somewhat appropriate/Neither appropriate nor inappropriate/Somewhat inappropriate/Very inappropriate)

**Q17. If the timing of the intervention message delivery felt inappropriate, why did you feel this?**
(Free text)

**Q18. How would you rate the content of the intervention messages?**
(Very relevant/Somewhat relevant/Neither relevant nor irrelevant/Somewhat irrelevant/Very irrelevant)

If response to Q18 is "somewhat irrelevant" or "very irrelevant":
**Q19. If the intervention message content felt irrelevant, why was this?**
(Free text)

**Q20. Did you find any of the intervention messages were particularly helpful?**
(Yes/Maybe/No)

If response to Q20 is "yes" or "maybe":
**Q21. How did the intervention messages align with your quit attempt?**
(Free text)

If response to Q20 is "yes" or "maybe":
**Q22. What messages and style of motivation helped you the most, and why?**
(Free text)

**Q23. Did you find any of the intervention messages were unhelpful?**
(Yes/No)

If response to Q23 is "yes":
**Q24. What was it about these intervention messages that you found unhelpful?**
(Free text)

**Q25. Please describe any positive aspects of your experience with this intervention.**
(Free text)

**Q26. Please describe any negative aspects of your experience with this intervention.**
(Free text)

**Q27. Do you have any other feedback you would like to give us about your experience with the smartwatch or the intervention?**
(Free text)

**Q28. Do you have any other questions about the study, or about how we will use your data?**
(Free text)
